# Assessment of the Medicare Advantage Risk Adjustment Model for Measuring Veterans Affairs Hospital Performance

**DOI:** 10.1001/jamanetworkopen.2018.5993

**Published:** 2018-12-14

**Authors:** Todd H. Wagner, Peter Almenoff, Joseph Francis, Josephine Jacobs, Christine Pal Chee

**Affiliations:** 1 Stanford University School of Medicine, Palo Alto, California; 2 Center for Innovation to Implementation, VA Palo Alto, Menlo Park, California; 3 Health Economics Resource Center, VA Palo Alto, Menlo Park, California; 4 Office of Secretary, Department of Veterans Affairs, Washington, DC; 5 Center of Innovation, Department of Veterans Affairs, Washington, DC; 6 Program for Quality Improvement/Patient Safety, School of Medicine, University of Missouri–Kansas City, Kansas City; 7 Office of Reporting, Analytics, Performance, Improvement, and Deployment, Department of Veterans Affairs, Washington, DC; 8 Department of Public Policy, Stanford University, Palo Alto, California

## Abstract

**Importance:**

Policymakers and consumers are eager to compare hospitals on performance metrics, such as surgical complications or unplanned readmissions, measured from administrative data. Fair comparisons depend on risk adjustment algorithms that control for differences in case mix.

**Objective:**

To examine whether the Medicare Advantage risk adjustment system version 21 (V21) adequately risk adjusts performance metrics for Veterans Affairs (VA) hospitals.

**Design, Setting, and Participants:**

This cohort analysis of administrative data from all 5.5 million veterans who received VA care or VA-purchased care in 2012 was performed from September 8, 2015, to October 22, 2018. Data analysis was performed from January 22, 2016, to October 22, 2018.

**Exposures:**

A patient’s risk as measured by the V21 model.

**Main Outcomes and Measures:**

The main outcome was total cost, and the key independent variable was the V21 risk score.

**Results:**

Of the 5 472 629 VA patients (mean [SD] age, 63.0 [16.1] years; 5 118 908 [93.5%] male), the V21 model identified 694 706 as having a mental health or substance use condition. In contrast, a separate classification system for psychiatric comorbidities identified another 1 266 938 patients with a mental health condition. The V21 model missed depression not otherwise specified (396 062 [31.3%]), posttraumatic stress disorder (345 338 [27.3%]), and anxiety (129 808 [10.2%]). Overall, the V21 model underestimated the cost of care by $2314 (6.7%) for every person with a mental health diagnosis.

**Conclusions and Relevance:**

The findings suggest that current aspirations to engender competition by comparing hospital systems may not be appropriate or fair for safety-net hospitals, including the VA hospitals, which treat patients with complex psychiatric illness. Without better risk scores, which is technically possible, outcome comparisons may potentially mislead consumers and policymakers and possibly aggravate inequities in access for such vulnerable populations.

## Key Points

**Question:**

Are current risk adjustment algorithms fair for comparing hospitals in the Veterans Affairs Health Care System with nonfederal hospitals?

**Findings:**

In this cohort study of 5.5 million patients who received care in the Veterans Affairs Health Care System, the Medicare Advantage risk adjustment system version 21 did not perform well in part because of inadequate psychiatric case mix adjustment.

**Meaning:**

The findings suggest that risk adjustment algorithms should expand their psychiatric case mix to prevent potentially misleading consumers and policymakers and aggravating inequities in access for vulnerable populations.

## Teaser

This cohort study of administrative data from the Veterans Affairs Health Care System assesses the applicability of using the Medicare Advantage risk adjustment system version 21 for comparing Veterans Affairs hospitals with nonfederal hospitals.

## Introduction

Consumers, purchasers, and policymakers want to compare hospitals on a wide array of performance metrics, including surgical complications or unplanned readmissions, measured from administrative data. The Centers for Medicare & Medicaid Services (CMS) publishes many metrics on their Hospital Compare website; hospitals affiliated with the Department of Defense (DoD) and the Department of Veterans Affairs (VA) also contribute data to CMS’s Hospital Compare website.^1^

The evolution of Hospital Compare is consistent with efforts to increase transparency and competition.^2^ For the VA hospitals, this push coincides with the passage of the $55 billion VA Mission Act, which supports veterans’ ability to choose where they get care. Although it seems reasonable to suggest that greater transparency and any ensuing competition will help patients, including veterans, some researchers have suggested that the VA hospitals do not compare well with commercial hospitals and that the VA hospitals should expand their role as purchasers.^3^ However, the Commission on Care, among others, concluded that the VA hospitals work well but need modernization so that they can be a learning health care system, as envisioned by the Institute of Medicine.^4,5^

Whether increasing transparency through hospital comparisons will motivate socially beneficial competition is unclear. The CMS publishes performance metrics on Hospital Compare, but the risk adjustment algorithms underlying these metrics are often unclear. The recent literature has questioned whether existing risk adjustment algorithms, including those used by the CMS to pay Medicare Advantage (MA) plans, accurately adjust for mental health comorbidities. For example, Montz and colleagues^6^ used commercial claim data from the Truven Health Analytics database to examine adjustment methods and payments to health plans. They found that the CMS risk adjustment algorithm missed 80% of individuals with a mental health or substance use diagnosis, leading to a systematic underpayment to plans for these individuals.^6^ Shrestha and colleagues^7^ followed up on this work by testing 21 algorithms for measuring mental health and substance use. They found notable variation in model performance but that substantial gains of as much as 10% were possible when analyzing commercial claims. Whether these findings translate to other hospitals that have a higher prevalence of patients with mental health and substance use problems is unknown.

We examined the applicability of using the Medicare risk adjustment model for comparing VA hospitals. We focused on the VA because it is a large safety-net institution that is under pressure to compare its hospitals with non-VA hospitals with the expectation that greater transparency will lead to improvements in access, quality, and cost. The importance of appropriate risk adjustment is highlighted by a recent Agency for Healthcare Research and Quality report,^8^ which found that veterans who receive care in the VA system are sicker than veterans who receive care elsewhere. However, whether existing risk adjustment models can level the playing field of statistical risk adjustment is unclear. In this study, we computed risk-adjusted costs for all VA patients in 2012 and then examined predicted costs for different subgroups, including patients with a diagnosis of diabetes, a mental health condition, or dementia. We used the CMS MA risk adjustment system version 21 (V21) because it is publicly available and has been used to adjust metrics published on CMS’ Hospital Compare website. In addition, it allowed us to examine whether technical improvements in the risk models were sufficient to overcome the deficiencies in the V21 model.

## Methods

### Study Population and Data

This study, performed from September 8, 2015, to October 22, 2018, included all 5.5 million veterans who received VA inpatient or outpatient care in 2012. We excluded patients who only used the VA for medications and who had no other VA use. We also excluded veterans who received care exclusively through other insurance programs. Veterans older than 65 years are selective in their use of VA and Medicare services.^9^ To avoid biased cost data, we included all VA and Medicare costs. For all participants, we obtained their VA and Medicare Part A, B, and D data. We excluded MA claims, which were not available, but noted that many veterans are enrolled in both VA and MA plans.^10^ The data included demographic information and *International Classification of Diseases, Ninth Revision* (*ICD-9*) diagnostics codes from inpatient and outpatient use. For VA costs, we used the VA Health Economics Resource Center (HERC) mean cost data for ambulatory care and inpatient care and VA managerial cost accounting data for pharmacy costs. We added payments from VA-purchased care as reported in the Fee Basis system. Annualized HERC and VA managerial cost accounting costs are similar,^11^ but the HERC costs are less prone to high cost outliers. To estimate Medicare costs, we used payments. This work was classified as a quality improvement effort, and we received a human subjects waiver from the VA Palo Alto Research and Development Committee and the Stanford University Human Subjects Office. This study followed the Strengthening the Reporting of Observational Studies in Epidemiology (STROBE) reporting guideline.

### Measures

For all VA patients, we obtained demographic information from the VA enrollment files. For each patient, we computed their risk score using the V21 model. For patients who spent less than 90 days in skilled nursing or long-term care, we used the V21 community score. For patients who spent more than 90 days in a skilled nursing or long-term care facility, we used the institutionalized V21 score. We included all diagnostic codes from both VA administrative data and Medicare claims data from the prior year (2011). Because many veterans also receive care from Medicare,^9^ the inclusion of diagnosis codes from Medicare claims data allowed us to capture the risk profiles of veterans who used both systems.

The V21 model creates 83 hierarchical condition categories (HCCs), including 4 for mental health and substance use (HCC54 drug/alcohol psychosis, HCC55 drug/alcohol dependence, HCC57 schizophrenia, and HCC58 major depressive, bipolar, and paranoid disorders). The V21 model was replaced by the V22 model with the implementation of *International Statistical Classification of Diseases and Related Health Problems, Tenth Revision (ICD-10)*. The 2 models are similar; V22 has 79 HCCs, although it includes the same 4 mental health HCCs that were used in V21.^12^ We also measured mental health comorbidities using the Psychiatric Case Mix System (PsyCMS)^13^; specific *ICD-9* and *ICD-10* coding for the PsyCMS can be found online.^14,15^

We computed the total cost of care for all veterans who used VA care in 2012. This total included all VA costs and payments by Medicare Parts A, B, and D. We included VA and Medicare costs to understand the full cost of care for these patients; analyzing only VA costs might bias the results by focusing on existing distortions in the marketplace.

### Statistical Analysis

 Data analysis was performed from January 22, 2016, to October 22, 2018. We regressed total costs on patients’ V21 risk scores. We used a linear model because the MA payment formula uses a linear additive model and estimated it using ordinary least squares.^16^ Using the regression estimates, we calculated predicted costs for all patients and compared predicted costs with actual costs. We did this by decile of predicted costs. This goodness-of-fit test showed how the risk adjustment model fits data by decile of predicted costs.

To explore whether the V21 risk adjustment could be improved, in a second set of regression models, we included indicators for 47 mental health conditions as measured by the PsyCMS.^13^ This grouping was developed to measure mental health and substance use in risk adjustment. We examined goodness of fit for all VA patients and for 3 subgroups: patients with diabetes, patients with a mental health diagnosis, and patients with dementia. The main comparisons of interest were how the patients with a mental health diagnosis, as measured by the V21, compared with all VA patients and those with diabetes. We chose diabetes because it is a common chronic condition that results in considerable costs. Dementia was included because it often requires custodial care, which the VA provides and Medicare does not cover. This comparison offers insights on whether risk adjustment models built on Medicare data are sufficient for comparing VA hospitals, which provide a different scope of services. We performed sensitivity analyses using general linear models (log link and a γ distribution) and a square root transformed ordinary least squares model. All analyses used a 2-sided test with *P* < .05 considered to be statistically significant.

## Results

### Sample Characteristics

A total of 5 472 629 VA patients (mean [SD] age, 63.0 [16.1] years; 5 118 908 [93.5%] male) were included in the study. Total spending on VA patients in 2012 was $67.4 billion, with $47.2 billion borne by the VA and the remainder paid by Medicare. Table 1 gives the characteristics of the 5.47 million VA patients and breakouts for those younger than 65 years and those 65 years or older. The mean (SD) cost of a VA patient was $12 126 ($30 090), with most of those costs borne by the VA (Table 1). The median total annual cost was $3955 (interquartile range, $1645-$10 185), which highlights inherent skewness in the costs. For patients 65 years or older, the total mean (SD) cost was $14 995 ($33 572), with $8074 ($27 801) paid by the VA and $6921 ($18 411) paid by Medicare. Medicare costs were considerably less for people younger than 65 years (Table 1).

**Table 1.** Sample Characteristics of VA Patients in 2012^a^

Characteristic	All VA Patients (N = 5 472 629)	VA Patients ≥65 y of Age (n = 2 390 568)	VA Patients <65 y of Age (n = 3 082 061)
Male	5 118 908 (93.5)	2 349 034 (98.3)	2 769 874 (89.9)
Age, mean (SD), y	63.0 (16.1)	76.9 (7.8)	52.2 (12.1)
Diagnostic codes as measured by V21			
Any diabetes diagnosis	1 211 089 (22.1)	685 101 (28.7)	525 988 (45.2)
Any MH/SA diagnosis	694 706 (12.7)	139 861 (5.9)	554 845 (18.0)
Diagnostic codes as measured by PsyCMS			
Any MH/SA diagnosis^c^	1 958 978 (35.8)	609 567 (25.5)	1 349 411 (43.8)
PTSD	571 654 (10.4)	125 652 (5.3)	446 002 (22.3)
Mood disorder	1 010 105 (18.5)	296 227 (12.4)	713 878 (32.9)
Serious mental illness	260 509 (4.8)	53 553 (2.2)	206 956 (14.8)
Substance abuse	981 144 (17.9)	233 533 (9.8)	747 611 (29.7)
Dementia	41 275 (0.8)	35 814 (1.2)	5461 (0.2)
Total costs, $			
Mean (SD)	12 126 (30 090)	14 995 (33 572)	9901 (26 872)
Median (IQR)	3955 (1645-10 185)	5095 (2280-13 548)	3195 (1288-8134)
VA costs, $			
Mean (SD)	8547 (26 344)	8074 (27 801)	8914 (25 149)
Median (IQR)	2613 (1117-6599)	2245 (1027-5587)	2964 (1214-7366)
Medicare costs, $			
Mean (SD)	3579 (13 867)	6921 (18 411)	987 (7946)
Median (IQR)	0 (0-708)	560 (0-4535)	0
V21 score, mean (SD)	0.73 (0.63)	0.91 (0.65)	0.59 (0.58)

Abbreviations: IQR, interquartile range; MH/SA, mental health and substance abuse; PsyCMS, Psychiatric Case Mix System; PTSD, posttraumatic stress disorder; VA, Department of Veteran Affairs; V21, Medicare Advantage risk adjustment system version 21.

^a^ Data are presented as number (percentage) of VA patients unless otherwise indicated.

### Fit of V21 Risk Model for VA Patients

Of the 5 472 629 VA patients, the V21 model identified 694 706 as having mental health or substance use HCCs. In contrast, the PsyCMS identified another 1 266 938 patients with mental health diagnoses. Table 2 gives the top 10 missed diagnoses ranked by their prevalence. The most common were nicotine dependence (509 926 [40.2%]), followed by depression not otherwise specified (396 062 [31.3%]), posttraumatic stress disorder (PTSD) (345 338 [27.3%]), and anxiety (129 808 [10.2%]).^13^

**Table 2.** Top 10 Psychiatric Case Mix System Mental Health Groups That Were Missed by Medicare Advantage Risk Adjustment System Version 21^a^

Mental Health Diagnostic Group	No. (%) of Patients (n = 1 266 938)
Nicotine dependence	509 926 (40.2)
Depression, not otherwise specified	396 062 (31.3)
Posttraumatic stress disorder	345 338 (27.3)
Anxiety	129 808 (10.2)
Organic other	120 324 (9.5)
Alcohol abuse	73 910 (5.8)
Adjustment reaction	71 128 (5.6)
Neurotic depression	63 390 (5.0)
Sexual dysfunction	59 026 (4.7)
General anxiety	44 130 (3.5)

^a^ Measured using diagnostic codes as specified by the Psychiatric Case Mix System.

Overall, the V21 model underestimated costs for patients with low costs and overestimated costs for patients with above-average costs except for the top decile (Table 3). However, when the sample was separated by diagnosis, the V21 model fit the diabetes population well across most of the deciles. For mental health, however, the V21 universally underestimated costs across every decile (Table 4). Overall, this resulted in an underestimate cost of $2314 per person (6.7%) for every person with a mental health diagnosis.

**Table 3.** Risk Adjustment Model Fit Among All VA Patients and Patients With Diabetes

Decile of Expected Cost	All VA Patients (N = 5 472 629)			Patients With Diabetes^a^ (n = 1 232 297)		
	Expected Costs, $	Mean Actual Costs, $	Difference, $ (%)	Expected Costs, $	Mean Actual Costs, $	Difference, $ (%)
1	2066	4219	−2153 (−104)	6213	7091	−878 (−14)
2	4521	5221	−700 (−15)	7548	7297	250 (3)
3	5706	6203	−498 (−9)	9002	9338	−336 (−4)
4	7372	7002	370 (5)	11 062	11 030	31 (0)
5	8962	9651	−689 (−8)	12 678	12 436	242 (2)
6	10 668	9595	1073 (10)	14 840	14 611	229 (2)
7	12 705	11 370	1335 (11)	17 427	16 800	627 (4)
8	15 319	13 349	1970 (13)	21 168	20 760	408 (2)
9	19 371	18 681	690 (4)	27 616	28 066	−450 (−2)
10	35 499	36 743	−1244 (−4)	49 231	52 938	−3707 (−8)

Abbreviation: VA, Department of Veterans Affairs.

^a^ Measured using diagnostic codes as measured by Medicare Advantage risk adjustment system version 21.

**Table 4.** Risk Adjustment Model Fit Among Patients With a Mental Health Condition and Dementia

Decile of Expected Cost	Mental Health^a^ (n = 1 958 978)			Dementia^a^ (n = 157 907)		
	Expected Costs, $	Mean Actual Costs, $	Difference, $ (%)	Expected Costs, $	Mean Actual Costs, $	Difference, $ (%)
1	2011	5349	-3337 (−166)	11 287	13 128	−1841 (−16)
2	4565	6994	−2429 (−53)	14 928	17 822	−2894 (−19)
3	5613	7379	−1766 (−31)	16 675	20 141	−3467 (−21)
4	7194	8830	−1636 (−23)	18 828	24 107	−5278 (−28)
5	8760	10 921	−2161 (−25)	21 087	27 179	−6092 (−29)
6	10 682	11 591	−909 (−9)	23 562	31 092	−7531 (−32)
7	12 442	13 276	−833 (−7)	26 788	35 853	−9065 (−34)
8	15 276	16 879	−1603 (−10)	31 380	42 682	−11 302 (−36)
9	19 488	21 467	−1979 (−10)	39 198	50 713	−11 515 (−29)
10	37 307	42 018	−4711 (−13)	63 859	76 673	−12 813 (−20)

^a^ Measured using diagnostic codes as specified by the Psychiatric Case Mix System.

### Improving the Model Fit for Mental Health

The Figure gives the mean difference between the predicted costs and actual costs by decile. A perfect fit across the deciles would be a horizontal line at zero. Adding the 47 PsyCMS condition categories improved the model fit for patients with a mental health condition, but the data showed that measurement issues remain, suggesting continued room for improvement. The results were not sensitive in the analytical model, although model fit statistics varied across models. The *R*^2^ was 0.12 in the ordinary least squares model, which is consistent with reported fit statistics for the V21 model.^16^ Inclusion of the 47 psychiatric condition categories improved the *R*^2^ to 0.14. Results were robust to the model choice; in the sensitivity analysis, the best-fitting model was the square root transformed model, which had an *R*^2^ of 0.19 with the V21 model and an *R*^2^ of 0.22 with the V21 model augmented with PsyCMS groups.

**Figure.**
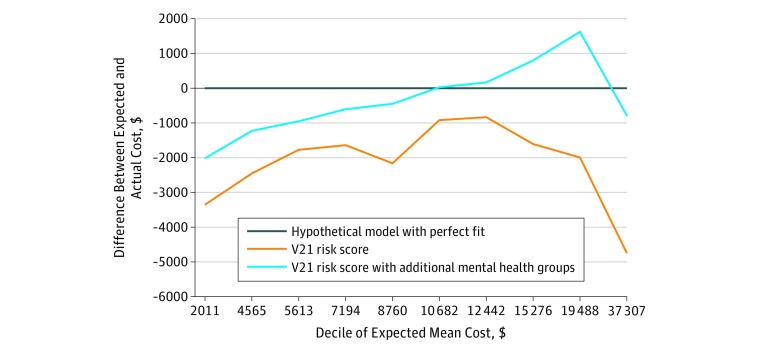
Improved Model Fit by Decile of Expected Cost V21 indicates Medicare Advantage risk adjustment system version 21.

### Dementia Care

Table 4 also gives the cost estimates for patients with dementia (n = 157 907). We used the institutionalized risk score for individuals who spent more than 90 days in skilled nursing or long-term care facilities. In this group, the V21 model underestimated costs by $1841 in the lowest cost decile, and this difference increased with each subsequent decile. In the highest cost decile, the difference between the expected and actual cost was $12 813.

## Discussion

Policymakers and consumers are eager to compare hospitals. Working to meet this demand, CMS provides a website that enables people to compare hospitals, including VA and DoD hospitals, on different performance metrics. Many comparisons focus on medical-surgical care, but it is possible to compare nursing homes, and CMS is rolling out additional comparisons, such as hospice. A motivating factor behind these websites is that greater transparency and more information will create incentives to improve quality of care by engendering competition. A critical assumption is that the risk adjustment algorithms used by Hospital Compare are sufficient to enable fair comparisons across performance metrics (eg, surgical complications, unplanned readmissions, or costs).

Our results highlight 2 important issues. First, when hospitals were compared, the V21 model did not perform well when the patients had mental health comorbidities. The CMS V21 model (and the subsequent V22 model) only accounts for 4 conditions related to mental health and substance use. Some important conditions for veterans, such as PTSD, are missing, whereas others of varying intensity are lumped together despite having different cost and utilization trajectories.^17,18,19^ Failing to adjust for mental health comorbidities extends beyond performance metrics for mental health care. Patients who have mental health comorbidities, including substance use disorders, have worse outcomes across a range of physical health conditions.^20,21,22,23^ Therefore, failing to adequately adjust for mental health comorbidities could skew a hospital’s performance metrics and create financial incentives that could have broad implications for how organizations target vulnerable populations.^24,25^ Technical improvements in the risk adjustment algorithms can help alleviate this problem. In addition, CMS recently released the V23 model, which includes more mental health categories, although it still does not include PTSD. Future research is needed to evaluate the V23 model and then apply the methods described by Shrestha and colleagues^7^ to optimize future iterations of the CMS risk adjustment model.

Second, risk adjustment models reflect the data on which they are built. The V21 does not adjust well for dementia care in the VA hospitals, largely because the V21 model was built on Medicare claims. The V21 model has a risk score for institutionalized patients, but even with these scores, it consistently underestimates costs among veterans using the VA hospitals, where custodial care benefits are more generous than those in Medicare. Showing that the V21 poorly predicts dementia care may seem obvious, but correcting this problem is more challenging than estimating an improved statistical model. If Hospital Compare is going to serve as a platform for comparing commercial, VA, and DoD hospitals, a risk adjustment model that is based on commercial, VA, and DoD data should be created. Otherwise, market distortions that are caused by differences in benefit generosity and risk selection may be perpetuated.

Comparing VA, DoD, and commercial hospitals is further complicated because these systems face incentives that can induce risk selection.^26^ For example, the VA’s mission is broader than just health care. The VA works to reduce homelessness and recidivism; the diversity of the VA’s work raises questions about whether the risk model should control for social determinants of health when performance metrics are being measured. This issue has been a matter of much debate.^27,28^ On the one hand, these social issues affect patients’ use of health care. On the other hand, it would be more expensive to treat homelessness through health care payments than directly through investments in housing. One possible solution is to build a Hospital Compare risk adjustment model that is not tied to the MA payment model.

Prior research, typically focused on specific conditions or populations, found that the VA provides equal or better quality care than non-VA hospitals.^29,30,31,32,33^ This finding differs from news reports that suggest that the quality of VA care is below that of non-VA hospitals. For example, the VA was recently criticized about nursing home ratings on Hospital Compare.^34^ It is possible that these discrepant findings are attributable to methodologic differences. The results of the present study suggest that use of risk adjustment when comparing the quality of care between VA and commercial hospitals is important. Comparing hospitals without adequate risk adjustment could generate false information that harms the VA and other safety net hospitals.^35,36^ Future research is needed to help us understand how sensitive the metrics on Hospital Compare are to different methods.

### Limitations

This study relies on data with different coding practices by VA and non-VA hospitals. One question is whether poor coding in the VA could have led to the results. The VA facilities receive capitated payments for each patient, and the practitioners are salaried; therefore, there are few incentives to code meticulously. In contrast, physicians in private practice, especially those with MA patients, have incentives that reward detailed coding.^37^ The question of bias attributable to poor coding in the VA hinges on whether VA practitioners are more likely to undercode mental health or physical health comorbidities. An article by Yoon and Chow^38^ suggests that VA practitioners are more likely to undercode mental health than other conditions. Thus, if mental health comorbidities are being under coded uniformly, our analysis is biased toward the null and these results are likely to be a conservative estimate.

Another limitation of this study is that we only tested the model fit for the V21 model. Other approaches that may work for disadvantaged populations include template matching,^39^ stratification, and peer comparisons,^40^ but their feasibility and practicality need to be tested. Commercially available risk adjustment algorithms may do a better job fitting VA data, but this would only further underscore the need to be careful when choosing a risk-adjusting algorithm because not all of them are useful for comparing health care systems.

The results generalize to the hospitals in the VA health care system. Variation is often seen across VA hospitals, and it is likely that individual VA hospitals differ in terms of the percentage of patients with mental health comorbidities, which could affect their ratings in Hospital Compare. It is unclear whether the results translate to the DoD or other safety-net hospitals, although it is likely that this problem persists in those settings given the work by Montz et al^6^ and Shrestha et al.^7^

## Conclusions

The findings suggest that current comparisons between VA and non-VA hospitals are flawed because the risk adjustment algorithms used to make patients comparable are not adequately controlling for mental health issues. Updating the risk adjustment model to account for more information on mental health, a process already under way at the CMS, is a step in the right direction. However, these risk scores may need to be developed based on a broader set of hospital data. Without such efforts, safety-net hospitals, such as the VA hospitals, may be penalized and consumers and policymakers may be misled.
